# Supplementary material for: Structure-Guided Engineering of a Family IV Cold-Adapted Esterase Expands Its Substrate Range
Source: Int J Mol Sci. 2022 Apr 24;23(9):4703. doi: 10.3390/ijms23094703 (PMC9100969; doi:10.3390/ijms23094703)
Supplement: Supplementary file 1 [file ijms-23-04703-s001.zip › ijms-1697184-supplementary.pdf]

Supporting information

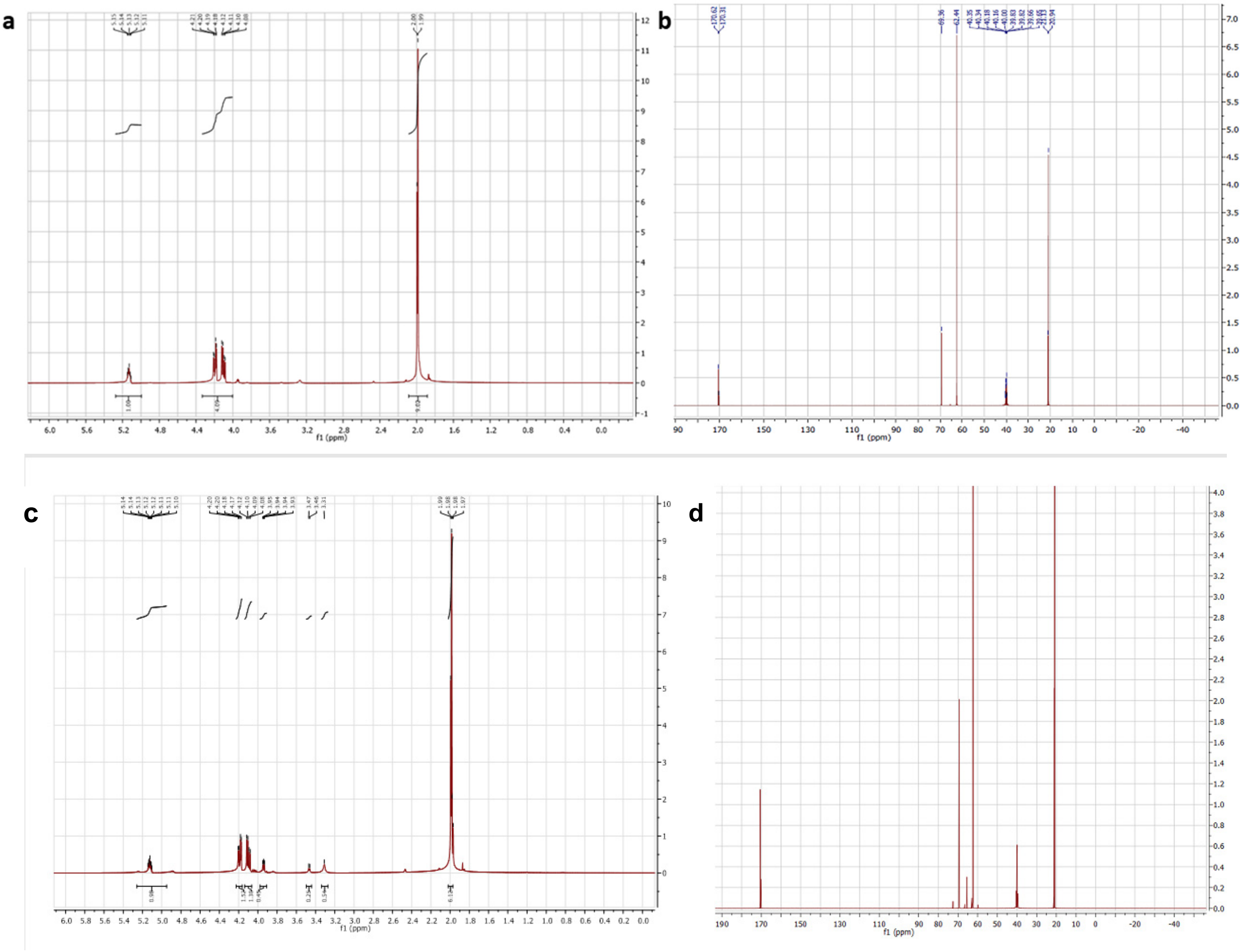

Figure S1. NMR analysis of triacetin and it breakdown product after incubation with EstN7. (a-b) The ^{1}H (a) and ^{13}C 1D NMR spectra of triacetin. (c-d) The ^{1}H (c) and (d) ^{13}C 1D NMR spectra of reaction products. Nuclear magnetic resonance spectra (^{1}H-NMR and ^{13}C-NMR) were recorded on JEOL 500 MHz spectrometers at ambient temperature operating at 500 and 125 MHz, respectively. Chemical shifts were reported in parts per million (ppm) and are referenced relative to DMSO at δ 2.50 ppm for DMSO-d_6.

Method. The enzyme regioselectivity towards triacetin was determined by NMR. The hydrolysis reaction was performed as stated previously, and at the end of the incubation period, the enzyme was removed from the reaction using affinity chromatography. 3 volumes of ethyl acetate were added for liquid portioning process to extract the hydrolytic products, the extraction was repeated at least 3 times. The collected ethyl acetate was totally evaporated and the remaining products were analysed by NMR using DMSO as a solvent. A blank sample was typically processed as described above.

Spectral Analysis. The overall structure of triacetin is shown in Figure 1a of the main manuscript, including the annotation of the central glyceryl unit. The ^{1}H-NMR spectrum

of triacetin (Figure S1a) shows two singlet peaks at 1.99 & 2.00 ppm chemical shift equivalent to 9 protons, which corresponds to the three methyl groups; a multiplet peak is observed at the range 4.08-4.21 ppm equivalent to 4 protons corresponding to the two (C-1 and C-3 of the glyceryl unit) methylene protons. The multiplet peak at the range 5.12-5.15 ppm equivalent to one proton correspond to the CH (C-2 of the glyceryl unit) proton. In addition, the ^{13}C-NMR spectrum of triacetin (Figure S1b) shows 5 peaks at 20.94, 62.44, 69.36, 170.31, 170.62 ppm, corresponding to CH_3, CH_2, CH, C=O, C=O groups. On the other hand, the ^{1}H-NMR spectrum of the reaction product (Figure S1c), shows additional multiplet peaks, compared to those observed for triacetin at the range 3.29-3.33, 3.45-3.48, and 3.92-3.96 ppm. In addition, the integration of the peaks at 1.98 and 1.99 ppm corresponding to the methyl groups, is equivalent to 6 protons, confirming the hydrolysis of one methyl group. The ^{13}C-NMR spectrum of the reaction product (Figure S1d) also shows a slightly deshielded peak at 65 ppm corresponding to either C-1 or C-3 of the glyceryl unit, which underwent hydrolysis. The hydrolysis at the central C-2 carbon of the glyceryl will give a symmetric reaction product resulting in significant deshielding of the peak corresponding to the C-2-H proton, which is not observed in the ^{13}C NMR spectra of the product (Figure S1d). The observed results confirm that hydrolysis occurred at either the terminal 1 or 3 carbon position.

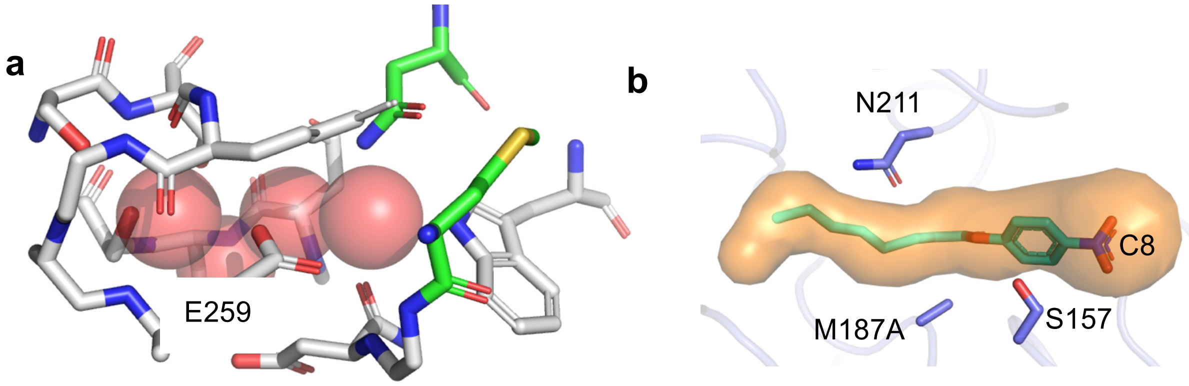

Figure S2. Opening the acyl pocket plug. (a) *In silico* modelling of acyl plug mutations and docking of substrate C8 for the M187A mutation. (b) Residues surrounding the water molecules in the second cavity beyond the plug. Residues coloured green are N211 and M187 that comprise the plug. E259 is highlighted as it is the only residue capable of making an ionic interaction with the water molecules.

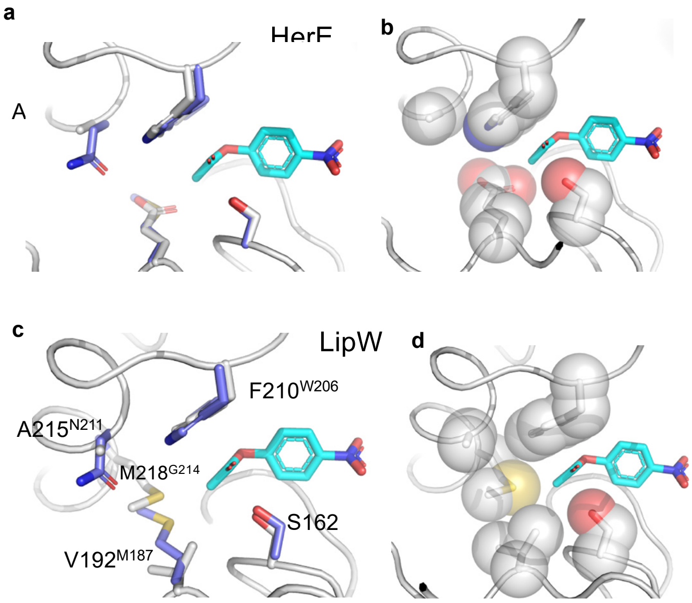

Figure S3. Comparison of EstN7 the acyl binding pocket with HerE (a,c) and LipW [1] (b,d) (PDB 3QH4). The pNP-C2 substrate is shown in cyan. Panels a and c show the structural overlap of EstN7 (blue) with its counterpart (grey). Panels c and d show the key residues involved in forming the acyl pocket plus for HerE and LipW, respectively.

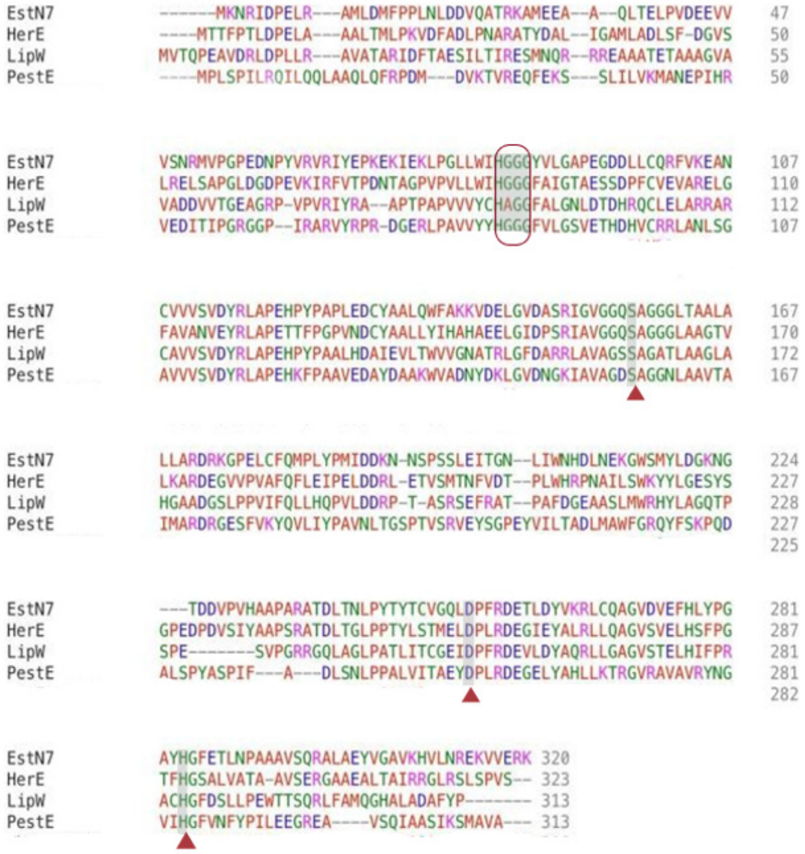

Figure S4: Multiple sequence alignment of EstN7 and close structural homologs; HerE (heroin esterase) from *Rhodococcus* sp. strain H1, a mycobacterial LipW (PestE from the thermophile *Pyrobaculum calidifontis*. Catalytic residues are indicated with triangles, while the oxyanion hole sequences are highlighted with a rounded square.

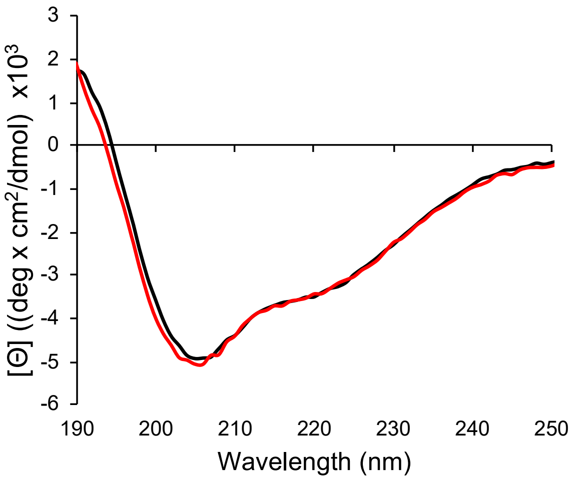

Figure S5. CD spectra of EstN7^{WT} (black) and EstN7^{N211A} (red) were measured at 70°C after temperature ramping.

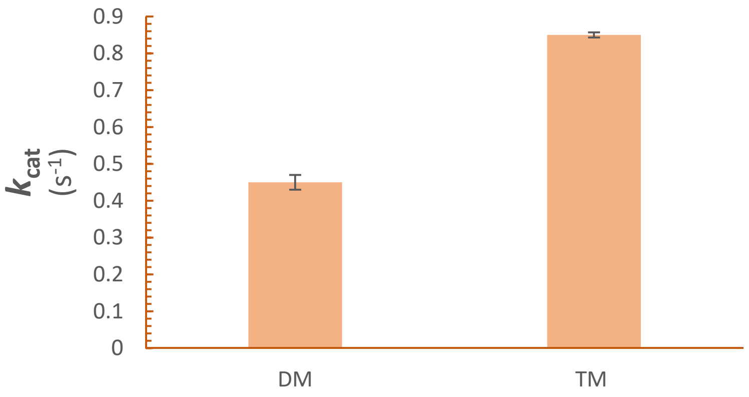

Supporting References

[1] McKary, M. G., Abendroth, J., Edwards, T. E., and Johnson, R. J. (2016) Structural Basis for the Strict Substrate Selectivity of the Mycobacterial Hydrolase LipW, *Biochemistry 55*, 7099-7111.

[2] Cumming, G., Fidler, F., and Vaux, L. D.(2007) Error bars in experimental biology, Journal of Cell Biology, 177, 7-11.
